# Case Report: Late Onset of Myelodysplastic Syndrome From Donor Progenitor Cells After Allogeneic Stem Cell Transplantation. Which Lessons Can We Draw From the Reported Case?

**DOI:** 10.3389/fonc.2020.564521

**Published:** 2020-10-14

**Authors:** Mirko Farina, Simona Bernardi, Lisa Gandolfi, Camilla Zanaglio, Enrico Morello, Alessandro Turra, Tatiana Zollner, Doriana Gramegna, Benedetta Rambaldi, Federica Cattina, Nicola Polverelli, Michele Malagola, Domenico Russo

**Affiliations:** ^1^Chair of Hematology, Unit of Blood Diseases and Stem Cell Transplantation, DPT of Clinical and Experimental Sciences, University of Brescia, ASST Spedali Civili di Brescia, Brescia, Italy; ^2^CREA Laboratory (Centro di Ricerca Emato-Oncologica AIL), ASST Spedali Civili di Brescia, Brescia, Italy

**Keywords:** donor cell myelodysplasia, transplant, leukemogenesis, immunosurveillance, immunosuppression, stem cells

## Abstract

**Background:**

Myelodysplastic syndromes and acute leukemias after allogeneic stem cell transplantation (allo-SCT) are mainly caused by recurrence of the primitive leukemic clones. More rarely, they originate from donor hematopoietic stem cells, developing the so-called donor cell leukemia (DCL) or myelodysplastic syndromes (DC-MDSs). DCL and DC-MDS can be considered as an *in vivo* model of leukemogenesis, and even if the pathogenetic mechanisms remain speculative, a genetic predisposition of donor progenitor cells, an altered host microenvironment, and the impairment of immune surveillance are considered the main causes.

**Case Presentation:**

We report a case of DC-MDS diagnosed 5 years after an allo-SCT from a matched related donor (patient’s sister) in a patient with Philadelphia chromosome-positive B-cell acute lymphoblastic leukemia (Ph+ B-ALL). The sex-mismatch allowed us to identify the donor cell origin. At the onset, the DC-MDS was characterized by chromosome seven monosomy and *NRAS*, *RUNX1*, and *BCOR* mutations. Because of a familiar history of colorectal neoplasia and the variant allele frequency (VAF) of *NRAS* mutation at the onset, this mutation was searched on germline DNA in both the donor and the recipient, but the result was negative. Moreover, after transplant (+4 months), the patient developed severe and long-lasting chronic graft-versus-host disease (cGVHD), requiring multiple lines of treatments. Because of the severe immunosuppression, recurrent infections occurred and, lately, the patient died due to septic shock.

**Conclusion:**

This case report highlights the need, whenever possible, to evaluate the donor origin of the posttransplant myelodysplasia and acute leukemias. The potential key role of the impaired immune surveillance and of long-lasting immunosuppression appears to be emerging in the development of this case of DC-MDS. Finally, this case reminds the importance to investigate the familiar genetic predisposition in donors with a familiar history of neoplasia.

## Introduction

Allogeneic stem cell transplantation (allo-SCT) is the most effective treatment for many hematologic diseases, being a curative approach. Unfortunately, posttransplant relapse is the first cause of transplant failure, and it occurs in at least 30–50% of patients with acute leukemia (AL) or myelodysplastic syndrome (MDS) (1, 2).

Most cases originate from the regrowth of the primitive leukemic clones. More rarely, they originate *de novo* from donor progenitor cells. Being an extremely rare event, very few cases are described in the literature and most of them are ALs, “donor cell leukemia” (DCL), while sporadically MDSs [“donor cell MDS” (DC-MDS)] (3, 4).

DCL and DC-MDS can be considered as an *in vivo* model of leukemogenesis, and even if the pathogenetic mechanisms remain speculative, a genetic predisposition of donor progenitor cells, an altered host microenvironment, and the impairment of immune surveillance are considered the main causes.

Although the historic division of DCL/DC-MDS into two groups remains useful (5), (1) a malignant clone inadvertently transferred to the recipient at the time of transplant, and (2) a group in which donor cells became malignant in the new host environment, it clearly appears that the DLC etiology is a multistep process, in which these two mechanisms could interact. Moreover, another crucial aspect to be taken into consideration is the importance of an effective and competent immune system in controlling the growth of a leukemic clone, whether it derives from a primitive damage/“hit” in the donor cells or by the influence of an altered host environment.

Here, we present a case of DC-MDS diagnosed 5 years after matched related allo-SCT in a patient with Philadelphia chromosome-positive B-cell acute lymphoblastic leukemia (Ph+ B-ALL).

## Case Presentation

**FIGURE 1:**
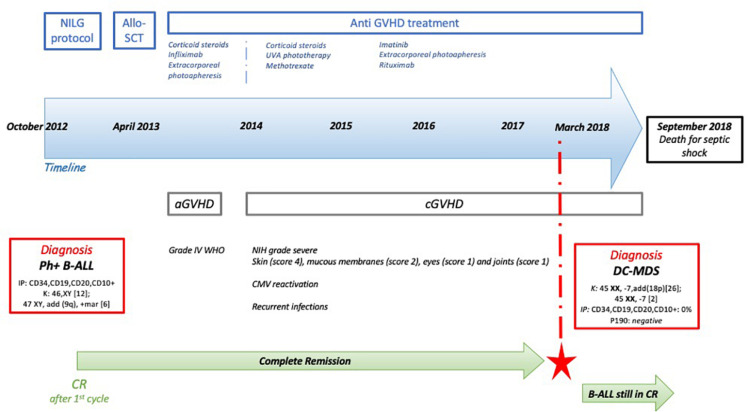
Patient’s timeline. At the top, the main treatments that the patient has received, with a focus on the anti-graft-versus-host disease (GVHD) therapies. Then, the blue arrow represents the timeline. In the red rectangles, the main diagnosis that the patient has received and the biological characteristics [Philadelphia chromosome-positive B-cell acute lymphoblastic leukemia (Ph+ B-ALL) and donor cell myelodysplastic syndrome (DC-MDS)]. In green, the persistence of the complete response (CR) over time, even after the DC-MDS onset (red star). K, karyotype; IP, immunophenotype.

In October 2012, a 49-year-old man was diagnosed with Ph+ B-ALL. At diagnosis, the patient presented with hyperleukocytosis and lymphoblasts expressing B-ALL immunophenotype (CD34+, CD19+, CD20+, CD10+). The Ph+ *t*(9; 22) translocation (Figure 1) resulted in a p190 protein (e1a2 transcript type), determining a high risk (HR) of disease relapse. The patient did not present any significant comorbidities, and he was enrolled in the NILG ALL10/07 trial (6) (Figure 1 and Supplementary Material 2), combining chemotherapy (idarubicin, cyclophosphamide, 6-mercaptopurine, dexamethasone, and intrathecal chemotherapy) and imatinib (800 mg/day).

The treatment was well tolerated, and the patient obtained a complete morphological, cytogenetic, and molecular response after induction therapy with no significant side effects. In April 2013, the patient underwent allo-SCT from a matched related donor (53-year-old patient’s sister, with no history of neoplasia or hematologic malignancies), considering the HR disease relapse. The conditioning regimen was based on cyclophosphamide 60 mg/kg for 2 days (4,800 mg total dose) and total body irradiation (total dose: 1,200 cGy), and he received methotrexate and cyclosporine-A (CyA) for graft-versus-host disease (GVHD) prophylaxis.

In the first 100 days after transplant, the patient developed a WHO grade 4 acute GVHD (aGVHD) (7) of the skin (stage III), gut (stage II), and liver (stage II). He was treated with steroids (6-methylprednisone 2 mg/kg per day), infliximab (four total doses), and extracorporeal photopheresis (ECP), obtaining complete remission.

Four months later, he developed severe chronic GVHD (cGVHD) (8) involving the skin (score 4), mucous membranes (score 2), eyes (score 1), and joints (score 1). The cGVHD was treated with UVA phototherapy, then with methotrexate, and lastly with imatinib, **ECP**, and rituximab. The posttransplant course was also characterized by several CMV reactivations and multiple infections of skin ulcers, complicated by recurrent sepsis and one episode of septic shock caused by ***Pseudomonas aeruginosa*** (July 2018).

During all the follow–up, the patient remained in continuous complete morphological/molecular remission with 100% CD34+ donor chimerism.

Five years after allo-SCT (January 2018), the patient presented with anemia [hemoglobin (Hb) 12.2 g/dl], thrombocytopenia [platelet (PLT): 71 × 10^9^/L], and hyperleukocytosis [white blood cell count (WBC): 20.45 × 10^9^/L] (Figure 1). MDS with excess blasts type 1 (MDS-EB1) was diagnosed according to the last WHO classification (9), with a very high International Prognostic Scoring System (IPSS)-revised score risk. The patient was still on cGVHD treatment with imatinib and tacrolimus. In the meantime, the primitive Ph**+** B-ALL was still in complete response (CR) (p190 negative; IF: CD34**+**, CD19**+**, CD10**+** = 0% of cells).

The cytogenetic analysis suggested the donor cell origin of MDS due to the female karyotype. Moreover, the karyotype was characterized by the presence of a monosomy of chromosome 7 (45 XX, −7, add(18p) [26]; 45 XX, −7q [2]). Conversely, no Ph+ metaphases were found. The chimerism on CD34+ cells (100% donor), together with the fluorescent *in situ* hybridization (FISH) for sex mismatch (361/361 XX), confirmed the donor cell origin (patient’s sister) of the MDS. At the time of DC-MDS, the patient presented severe lymphopenia (CD4+ : 80/μl; total lymphocytes: 500/μl). In parallel, the patient’s sister has been periodically evaluated, and her peripheral blood counts have always been in range.

A gene panel deep sequencing has been performed at DC-MDS diagnosis to analyze some genes known to be related to myeloid malignancies (Supplementary Figure 1). All the promoters and coding sequences of the genes were considered for the analysis. Next-generation sequencing (NGS) analysis revealed *NRAS* [variant allele frequency (VAF) 51%], *RUNX1* (VAF 62.2%), and *BCOR* (VAF 44.7%) mutations. Notably, the variants detected were not known to be associated with malignancies or other diseases. Considering the VAF of *NRAS* and the familial history for neoplasia (mother and a sister, not the transplant donor, with colorectal cancer), we investigated the presence of a familiar genetic predisposition for the development of hematologic and solid malignancies. To this purpose, the patient and the donor were enrolled in the NEXT-FamlY clinical trial (NCT03058588) (10). This is a national multicentric study focused on the analysis of known and unknown mutations related to familiar predisposition to hematologic malignancies. The germline DNA of both the donor and the recipient resulted negative for the *NRAS* mutation.

Unfortunately, because of the patient’s conditions compromised by the recurrent infections and GVHD complications, any treatments for the MDS could not be given and the patient died of septic shock a few months after the onset of DC-MDS (September 2018).

## Discussion

DCL and DC-MDS are very rare events that may occur in transplanted patients (3, 5, 11–18). In the literature, they usually onset after a median of 20 months (12, 17), with a maximum of 193 (12) months from transplant. They account for about 0.1% of the transplants, with an estimated incidence between 80.5 and 476 cases per 100,000 transplant recipients (11, 17, 18).

The first DCL case was described by Fialkow et al. (19) in 1971. Since then, few cases have been described (5, 11–18), even if they appear to be steadily increasing in the last years as result of more routine and sensitive post-allo-SCT chimerism tests or, possibly, of the increasing number of older donors who are more likely to carry clonal hematopoiesis (20–22).

The umbilical cord seems to bear a potential higher risk for DLC than other stem cell sources (23). Conversely, no differences have been described between bone marrow or peripheral blood sources. In addition, no relationship between DCL and the use of a related or unrelated donor has been observed. Therefore, in this reported case, since the patient received peripheral stem cells from his sister, he did not present a higher risk of DCL, considering the source of stem cells.

Cyclophosphamide with total body irradiation (Cy + TBI) is the conditioning regimen reported to be more frequently associated with DCL development (24). This is the case of our patient who received Cy + TBI before developing DC-MDS 60 months later.

In this presented case, the sex-mismatch easily allowed the recognition of the donor cell origin—thanks to the karyotype and the FISH analysis for gonosomal. Conversely, the cytogenetic analysis is not helpful for detecting the donor origin of MDS/leukemias in sex-matched transplants, for whom the DCL diagnosis is more difficult and often underestimated. In these patients, variable number of tandem repeats or short tandem repeat (STR) analysis should be used to detect DCL/DC-MDS, together with the study of chimerism on CD34+ cells in peripheral blood.

At DC-MDS onset, the patient presented with chromosome seven monosomy, which is the most common cytogenetic alteration observed in DC-MDS and DCL (12). Despite being the most frequently cytogenetic alteration, its impact on patients’ outcome is not yet very well established due to the low number of cases described. Recently, the presence of additional somatic mutations has been reported to influence the outcome of MDS patients with chromosome 7 alterations (25).

***NRAS*, *RUNX1***, and ***BCOR*** genes were mutated as shown by NGS analysis. ***NRAS*** and ***RUNX1*** are among the most frequently mutated genes in patients with MDS and monosomy 7/deletion 7q (25), and they were associated with adverse prognosis in previous studies (26, 27). Interestingly, in our case, ***NRAS*** presented a VAF suspicious for being a germline mutation. In addition, the patient and the related donor presented a familiar history for neoplasia (colorectal cancer in the mother and the sister), and the so-called “blend pedigree” have been described in literature in the last years (10, 28).

Therefore, germline DNA from epithelial buccal cells of the donor and the patient was sequenced using traditional Sanger methods. However, neither the donor nor the recipient presented the variant of *NRAS* on germline DNA. Nevertheless, the evaluation of the donors’ clinical history investigating the presence of a possible pedigree suspicious for familiar neoplasms should always be strongly recommended. That is in order to avoid the use of donor cells that may already present a genetic familiar predisposition to develop hematological malignancies after allo-SCT.

As expected, DCL and DC-MDS prognosis is poor (11), with a median overall survival of 11 months (0–91) (11). In our case, the poor condition of the patient did not allow us to employ any treatment and the death of the patient occurred 8 months after the onset of DC-MDS due to septic shock.

Although several hypotheses have been made, the underlying pathogenetic mechanisms for DCL/DC-MDS developing remain unknown and speculative (12, 13). The main mechanisms proposed included occult leukemia in the donor, alteration of the niche, immune-mediated phenomenon, infections (29), toxicities of posttransplant therapies, and GVHD (30–35). The long latency, together with the fact that the donor remains free from malignancies, in addition to the absence of *NRAS* variants on germline DNA, supports the hypothesis that either the host bone marrow microenvironment or the immunosuppression could play a pathogenetic role in this presented case. On one hand, the primitive Ph+ ALL and the subsequent chemotherapies should have determined an altered microenvironment, which plays a crucial role in leukemogenesis (36–39). Nevertheless, the long story of cGVHD and the large use of different immunosuppressive therapies could possibly have favored the onset of DC-MDS, though an impaired immunosurveillance. Indeed, cGVHD (40, 41) and GVHD treatments have a profound suppressive effect on T-lymphocytes, which are the main responsible for the immune response against tumor-specific antigens (42). In fact, our patient presented severe lymphopenia at the time of DC-MDS diagnosis. Furthermore, the immunosuppressive effects of cGVHD and of the related treatments worsen the T-immunodeficiency caused by allo-SCT for itself (13, 43). In addition to this scenario, the recurrent infections, which required hospitalizations and, ultimately, led to the death of the patient, strongly support the hypothesis of an impaired immunosurveillance in this presented case.

## Conclusion

Despite being rare events, DC-MDS and DCL may occur as late complications after allo-SCT. They are an intriguing phenomenon, and they were probably underdiagnosed for many years. Therefore, searching for the donor cell origin should be performed in all patients relapsing after allo-SCT. The new molecular technologies for chimerism monitoring made easier and more reliable the DCL diagnosis even when the donor origin could not be easily detected, such as in sex-matched cases.

Donor cell-myelodysplastic syndrome and DCL represent an *in vivo* leukemogenesis model to investigate the underlying pathogenetic mechanisms, which, so far, remain speculative. Beyond the hypothetical pathogenetic mechanisms, the setting of cGVHD and related immunosuppressive treatments may allow DCL development through an impaired immunosurveillance. Conventional and new interesting approaches are available and should be considered to monitor the immune reconstitution and the development of different competent lymphocyte populations (44–46).

Finally, considering that the DCL and DC-MDS may be a consequence of a genetic predisposition, the familiar history for neoplasia of the donors has to be investigated. When it is present, searching for DNA germline mutations both in the recipient and in the donor is mandatory to avoid the use of donor cells that may have a genetic predisposition to develop DCL/DC-MDS after allo-SCT.

## Data Availability Statement

The raw data supporting the conclusions of this article will be made available by the authors, without undue reservation.

## Ethics Statement

The studies involving human participants were reviewed and approved by Comitato Etico di Brescia. The patient provided their written informed consent to participate in this study and for the publication of this case report.

## Author Contributions

MF, SB, MM, and DR designed the study. MF, SB, MM, TZ, LG, DG, EM, NP, AT, CZ, and BR collected and analyzed the data. SB and CZ performed the laboratory NGS analysis. MF, SB, MM, and DR wrote the manuscript. All authors gave their final approval before submission and contributed to the article and approved the submitted version.

## Conflict of Interest

The authors declare that the research was conducted in the absence of any commercial or financial relationships that could be construed as a potential conflict of interest.

## Abbreviations

allo-SCT: allogeneic stem cell transplantation
ALL: acute lymphoblastic leukemia
AML: acute myeloid leukemia
DC-MDS: donor cell myelodysplastic syndrome
DCL: donor cell leukemia
FISH: fluorescent *in situ* hybridization
cGVHD: chronic graft-versus-host disease
aGVHD: acute graft-versus-host disease
GVHD: graft-versus-host disease
MDS: myelodysplastic syndrome
Ph+ B-ALL: Philadelphia chromosome-positive B-cell acute lymphoblastic leukemia
STR: short tandem repeat
VAF: variant allele frequency
WBC: white blood cell count
PLT: platelets
Hb: hemoglobin.
