# Supplementary material for: Case Report: Late Onset of Myelodysplastic Syndrome From Donor Progenitor Cells After Allogeneic Stem Cell Transplantation. Which Lessons Can We Draw From the Reported Case?
Source: Front Oncol. 2020 Oct 14;10:564521. doi: 10.3389/fonc.2020.564521 (PMC7591784; doi:10.3389/fonc.2020.564521)
Supplement: Supplementary file 1 [file Data_Sheet_1.PDF]

ASXL1	BCOR	NRAS	TP53	RUNX1
CEBPA	FLT3	EZH2	IDH1	IDH2
NPM1	DNMT3A	TET2	CBL	KRAS
ETV6	SF3B1	SRSF2	U2AF1	ZRSR2
GATA2	TERT	TERC	SRP72	ANKRD26

Supplementary Material 1: NEXT-Famly gene panel. All the promoters and coding sequences of the genes were considered for the analysis.
