# Supplementary material for: Case Report: Late Onset of Myelodysplastic Syndrome From Donor Progenitor Cells After Allogeneic Stem Cell Transplantation. Which Lessons Can We Draw From the Reported Case?
Source: Front Oncol. 2020 Oct 14;10:564521. doi: 10.3389/fonc.2020.564521 (PMC7591784; doi:10.3389/fonc.2020.564521)
Supplement: Supplementary file 2 [file Data_Sheet_2.PDF]

CNS prophylaxis

Standard TIT is administered during induction/consolidation/maintenance. CR patients continue CNS prophylaxis until relapse in any site or allogeneic/autologous SCT.

TIT (triple IT therapy) with methotrexate 12.5 mg, cytarabine 50 mg, prednisone 40 mg on:

- days 1 and 15 of induction/consolidation cycles 1, 2 and 8;
- day 1 of consolidation cycles 4 and 6;
- day 1 of maintenance cycles 2, 3, 4 and 5.

(total no. 12)

Patients with CNS involvement at diagnosis:

TIT with methotrexate 15 mg, cytarabine 75 mg, prednisone 40 mg bi-weekly until CNS remission, followed by bi-weekly x2 and monthly x12 (except during consolidation cycles 3, 5 and 7).

Induction/Consolidation Therapy

It includes 3x targeted-infusion methotrexate 5 g/m^2, age >55 years (methotrexate reduced to 1.5 g/m^2), radiatiotherapy (Gy 36) after cycle 3 for CT-PET residual viable mediastinal mass > 2 cm. Patients with BM involvement not in CR after cycles 1-2 and those with mediastinal mass unmodified after chemo and radiatiotherapy are off study. For CR evaluation bone marrow is checked on days 28 and/or 56 in BM positive cases and CT-PET in patients with mediastinal mass after cycle 3. Consolidation cycles are administered at 21-28 day intervals.

- *Cycle 1*: prednisone 20 mg/m^2/bd PO (per os) on days -5 to -1, cyclophosphamide 300 mg/m^2/d IV (intravenous) on days -3 to –1 (pre-induction); idarubicin 12 mg/m^2/d IV on days 1 and 2, vincristine 1.4 mg/m^2/d (max. 2 mg) on days 1, 8, 15 and 22, L-asparaginase (E.Coli) 3.000 U/m^2 IV on days 8, 10, 12, 15, 17 and 19, dexamethasone 5 mg/m^2/bd IV on days 1-5, 15-19, G-CSF from day 5 (induction).
- *Cycle 2:* idarubicin 12 mg/m^2/d IV on day 1, cyclophosphamide 1000 mg/m^2 IV on day 1, dexamethasone 5 mg/m^2/bd IV/PO on days 1-5, cytarabine 75 mg/m^2/d IV/SC (subcutaneous) on days 2-5 and 9-12, 6-mercaptopurine 60 mg/m^2/d PO on days 1-14, G-CSF from day 7.
- *Cycles 3,7*: methotrexate 5 g/m^2/d IV on day 1 (24-h infusion, folinic acid rescue), cytarabine 2 g/m^2/bd IV on d ays 3 and 4, G-CSF from day 8 (collection/cryopreservation of autologous blood stem cells at cycle 3). Radiatiotherapy (Gy 36) after cycle 3 for CT-PET residual viable mediastinal mass > 2 cm.
- *Cycles 4,6:* idarubicin 12 mg/m^2/d IV on day 1, cyclophosphamide 1000 mg/m^2 IV on day 1, vincristine 1.4 mg/m^2/d (max. 2 mg) IV on days 1 and 8, dexamethasone 5 mg/m^2/bd IV/PO on days 1-5, cytarabine 75 mg/m^2/d IV/SC on days 2-5 and 9-12, 6-mercaptopurine 60 mg/m^2/d PO on days 1-14, G-CSF from day 7.
- *Cycle 5:* methotrexate 5 g/m^2/d IV on day 1 (24-h infusion, folinic acid rescue), L-asparaginase (E. Coli) 10.000 U/m^2 IV on days 3 and 8.
- *Cycle 8:* idarubicin 10 mg/m^2/d IV on days 1 and 8, vincristine 1.4 mg/m^2/d (max. 2 mg) IV on days 1 and 8, cyclophosphamide 300 mg/m^2/d IV on days 1-3, dexamethasone 5 mg/m^2/bd IV/PO on days 1-5, prednisone 20 mg/m^2/bd PO on days 8-12, G-CSF from day 10.

Variations for age >55 years (all):

- *Cycles 3, 7*: methotrexate 1.5 g/m^2.
- *Cycle 5*: methotrexate 1.5 g/m^2.

Supplementary material 2: NILG ALL10/07 protocol scheme (Bassan et al., Blood 128,22,12 – 2016)
